# Four minutes for a patient, twenty seconds for a relative - an observational study at a university hospital

**DOI:** 10.1186/1472-6963-10-94

**Published:** 2010-04-09

**Authors:** Gerhild Becker, Dorothee E Kempf, Carola J Xander, Felix Momm, Manfred Olschewski, Hubert E Blum

**Affiliations:** 1 Department of Internal Medicine II, University Medical Center Freiburg, Freiburg, Germany; 2 Palliative Care Research Group, University Medical Center Freiburg, Freiburg, Germany; 3 Department of Radiotherapy, University Medical Center Freiburg, Freiburg, Germany; 4 Department of Medical Biometry and Statistics, University Medical Center Freiburg, Freiburg, Germany

## Abstract

**Background:**

In the modern hospital environment, increasing possibilities in medical examination techniques and increasing documentation tasks claim the physicians' energy and encroach on their time spent with patients. This study aimed to investigate how much time physicians at hospital wards spend on communication with patients and their families and how much time they spend on other specific work tasks.

**Methods:**

A non-participatory, observational study was conducted in thirty-six wards at the University Medical Center Freiburg, a 1700-bed academic hospital in Germany. All wards belonging to the clinics of internal medicine, surgery, radiology, neurology, and to the clinic for gynaecology took part in the study. Thirty-four ward doctors from fifteen different medical departments were observed during a randomly chosen complete work day. The Physicians' time for communication with patients and relatives and time spent on different working tasks during one day of work were assessed.

**Results:**

374 working hours were analysed. On average, a physician's workday on a university hospital ward added up to 658.91 minutes (10 hrs 58 min; range 490 - 848 min). Looking at single items of time consumption on the evaluation sheet, discussions with colleagues ranked first with 150 minutes on average. Documentation and administrative requirements took an average time of 148 minutes per day and ranked second. Total time for communication with patients and their relatives was 85 minutes per physician and day. Consequently, the available time for communication was 4 minutes and 17 seconds for each patient on the ward and 20 seconds for his or her relatives. Physicians assessed themselves to communicate twice as long with patients and sevenfold with relatives than they did according to this study.

**Conclusions:**

Workload and time pressure for physicians working on hospital wards are high. To offer excellent medical treatment combined with patient centred care and to meet the needs of patients and relatives on hospital wards, physicians should be given more time to focus on core clinical tasks. Time and health care management solutions to minimize time pressure are required. Further research is needed to assess quality of communication in hospital settings.

## Background

Communication in hospitals matters. A trusting relationship with patients and their families is built on open, honest communication. However, today's health care environment makes good communication among patients, families, and caregivers harder and harder to achieve. As the walkouts in German hospitals emphasised at the beginning of 2006, hospital physicians face mounting demands on their time in today's hospital environment. Hospital stays are shorter, medical care is more technologically complex, resources are constrained, and there is a growing need for patients and families to have more information about, and involvement in, care decisions. Physicians complain that increasing administrative requirements for health care delivery, documentation tasks and even enhancing possibilities in medical examination techniques claim their energy and encroach on the time spent on communication with patients [1-3]. In addition, an increase in administrative tasks has been shown to be associated with increasing time pressure and low job satisfaction [4]. The time for communication and personal contact between physicians and patients seems to be an increasingly valuable resource [5,6].

Physicians spend time in face-to-face contact with patients gathering information, carrying out medical interventions, planning medical treatment, doing administrative work, and maintaining their knowledge base. But how much time do physicians in hospitals really spend on communication with patients? And how much time do they spend on communication with relatives? Studies researching quantity and/or quality of communication between physicians and patients on hospital wards are scarce.

In this context, our study has aimed to investigate how much time physicians on a hospital ward really spend on the communicating with patients and their relatives. External time measurements were compared with physicians' self-assessment. To find the potential predictors of communication times with patients and relatives, the physicians' gender and years of professional experience were assessed. In addition, we wanted to find out how much time physicians spent on other working tasks such as medical treatments, discussions with colleagues, or administrative requirements.

## Methods

### Setting and sample

The study took place in the Medical University Center Freiburg, Germany, an acute care and teaching hospital with more than 1700 beds on 110 wards and an average of 55.000 inpatients per year. The hospital has a computerised test-ordering and results-viewing system and an electronic discharge summary system, but relies on paper medical records for other functions. All departments and wards belonging to the clinics of internal medicine, surgery, radiology, neurology, and to the clinic for gynaecology at the hospital took part in the study. Before a large-scale study was conducted, the method was piloted on eight wards within five different departments at the clinic for internal medicine.

### Measurement tool

**Figure 1:**
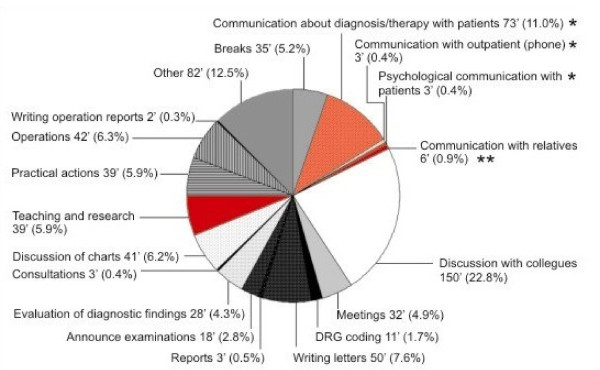
**Areas of work and time measurements**. * Total communication time: 4.4' per patient. ** Communication time with relatives: 20" per patient.

To assess the time physicians spent on different tasks on a ward, a multidimensional work task classification tool was used. The measurement tool was based upon a prepared classification system which had been developed by the research group and modified according to the results of the pilot period. The classification system comprised 19 areas of work (see Figure 1) which can be grouped into 6 categories (see Table 1).

**Table 1:** Distribution of the physicians' activities observed during the study period

Category	minutes per day	(% of working time)
**I Communication**		**40, 4%**
communication with patients about diagnosis/therapy	73	11,0%
communication with patients about psychosocial issues	3	0.4%
communication with relatives	6	0.9%
communication with outpatients (phone)	3	0.4%
discussion with colleagues	150	22.8%
meetings (regular discussion sessions on medical concerns)	32	4.9%
		
**II Patient Care**		**23.1%**
discussion of charts	41	6.2%
evaluation of diagnostic findings	28	4.3%
consultations (medical advice for patients on other wards)	3	0.4%
practical actions/medical activities	39	5.9%
operations	42	6.3%
		
**III Clinical documentation**		**11.2%**
writing (discharge) letters	50	7.6%
announce examinations	18	2.8%
Reports	3	0.5%
writing operation reports	2	0.3%
		
**IV Administrative documentation**		**7.6%**
DRG Coding	11	1.7%
		
**V Teaching and research**	39	**5.9%**
		
**VI Other activities**		**17.7%**
Breaks	35	5.2%
other (including walking times etc.)	82	12.5%
		
**Sum**		**100%**

The measurement tool was tested in a pilot period and revised on the basis of the feedback of the physicians and the observers after the pilot period.

Four subcategories (completing transportation orders, preparation of documentation forms, documentation of medication, documentation of findings) were removed and integrated in other categories to make the extrinsic time measurement more practicable. Walking times and social activities were classified as others.

If the doctor was conducting two tasks at the same time, the task he had started first was tested and assigned to the appropriate category. There was only one exception: when the patient or the intern started a conversation during the treatment the observer was instructed to change the code and to clock the communication time.

### Data collection

To minimize potential observer biases a pilot period was conducted. Eight physicians on eight different wards were observed during one work day each by two data collectors simultaneously, but independently. The congruence between the observations was 94% (range 87% - 98%).

During the study period, all measurements were done by a fourth year clinical medical student who had been specifically trained to code and time all activities performed by physicians on a ward. In addition, the student was briefed on confidentiality and professional working practices. She was not part of the medical establishment.

Over a study period of six months, the daily work of 34 physicians working on 36 different wards was researched. Data of 34 different wards and data of 32 physicians working on these wards could be included. Observation data of two wards had to be excluded because one ward was used for out-patient care at the observation day. Observation data of the other ward had to be excluded because the physician left the ward after two hours of observation as it was the morning after his night duty.

The wards and physicians to be measured were randomly chosen every day (simple randomization by blinding and numbering the wards and throwing the dice). If there were several interns working on the same ward, the intern who was responsible for the ward was tested. One workday on a ward legally lasts 8 hours. All 34 physicians observed were interns and were shadowed during one whole workday, from the beginning of their work in the morning until they left the ward in the evening.

After the measurements all physicians examined were asked to fill in a self-assessment-sheet to assess their estimations concerning length of time they spend on different fields of work each day. In addition, they were asked to rate their satisfaction with their work and their contentment with time for communication with patients and relatives on a 6-point Likertscale, ranging from 1 (very good) to 6 (unsatisfactory). We also assessed the physicians' gender and years of professional experience. 31 of 32 physicians observed filled in the self-assessment-sheet (data shown in Table 2).

**Table 2:** Demographic data according to self-assessment-sheets (31/32 physicians)

n	31
gender - m : f	17 : 14
time of professional life [months]	30 (range: 4 - 192 months)
not surgical : surgical	19 : 12

Because the tasks of physicians working in the surgical departments vary according to daily operation times, one randomly chosen physician working in the department of general surgery was tested during a period of five successive workdays to get reliable cross-sectional data.

### Data analysis

All measurements were collected in a database and checked by two independent persons. Average times for all areas of work were calculated. We tried to include possible predictive factors for communication time by univariate t-tests. As no significant predictive factors were found, we refrained from multivariate testing.

A two-tailed paired t-test was performed for the significance of the differences between self-assessed and measured time required for communication and documentation.

The dichotomization of the communication time was done by calculating the median. All data beyond the median were defined as 'short communication time', all data above the median were defined as 'long communication time'.

Furthermore, we calculated and tested the Pearson correlation of physicians' contentment concerning their work and the communication with patients and relatives with their gender and years of professional experience.

The data were analysed using the statistic software package SPSS 13.0 for windows^®^. Charts and diagrams were created with Microsoft excel 2000^®^, SigmaPlot 2002 for windows^® ^and CorelDraw 10^®^.

### Ethical approval

The study was approved by the Ethics Committee of Freiburg University Hospital.

## Results

### Demographic data

Daily activities of 32 physicians working on 34 different wards were measured and documented. The demographic data of 31 physicians taking part in the study are depicted in Table 2. One physician did not fill in the self-assessment-sheet. Two physicians changed the ward within the period of measurement and thus have been measured twice while working on different wards. In total, 374 working hours could be accounted for by the various codes which are 100% of observed working time.

On most of the wards two (or even more) physicians were working at the same time, sometimes on complex shift systems. We always observed the physician who was primarily accountable for the ward. As the second physician of a ward was often responsible for other tasks like ultra sound or operation theatre we decided to calculate the physicians' time per patient by including all patients on the ward for which the observed physician was responsible. Details on the wards, patients and weekdays of our study are depicted in Table 3.

**Table 3:** Number of wards included and number of weekdays assessed

departments *non surgical*	wards [no]	departments *surgical*	wards [no]
Cardiology	3	Accidental/Orthopedic Surgery	3
Gastroenterology	3	Chest surgery	1
Gynaecology	4	General/Abdominal Surgery	2
Haematooncology	5	Heart/Vascular Surgery	2
Infectiology	1	Urology	2
Neurology	2		
Nephrology	1		
Pneumatology	1		
Rheumatology	2		
Radiooncology	2		

**weekdays**	**[no]**	**wards**	

Monday	4	private:	6
Tuesday	9	governmental:	25
Wednesday	6	mixed:	3
Thursday	10	**inpatients **on wards during	618
Friday	5	study period	(mean = 18 pts/ward; range= 6-23)

### Time measurements

The time measurements revealed an average of 658.91 minutes (= 10 hrs 58.91 min; range 490 - 848 min) working hours per physician and day with an average time for breaks of 35 minutes. Looking at single items of time consumption, discussions with colleagues ranked first with 150 minutes (2 hrs 30 min) in average and accounted for 22.8% of coded time. Documentation including coding according to Diagnosis Related Groups (DRGs), (operation)reports, letters, administrative requirements and others ranked second and took an average time of 148 minutes (2 hrs 28 min = 21.5% of work hours). The average time spent on performing each task across all working hours is shown in Figure 1.

Total time for communication with patients was 79 minutes per physician per day. Total time for communication with relatives was 5 minutes and 54 seconds per physician per day (range 32.23 - 147.45 min for patients and 0 - 24.2 min for relatives, respectively). Communication time included time for diagnostic and therapeutic conversations with patients, time for communication on psychosocial issues with patients and time for dialogues with out-patients on the telephone. Divided by the number of patients on the particular ward, we calculated an average communication time of 4 minutes and 17 seconds per day per patient and 20 seconds per day per patient's relatives.

Predictive factors for a short communication time could not be found, even by univariate testing. Gender, years of professional experience, satisfaction with work, total working time per day, total documentation time per day, number of patient admissions or check outs, number of patients on the ward or patients with private or compulsory health insurance or surgeons versus non-surgical physicians did not have any effect on our results. Moreover, no significant differences concerning communication times with patients or relatives were found between physicians working on surgical wards and physicians working on non-surgical wards.

### Self-assessment

The response rate of self-assessment was 96%. The physicians who took part in our study were convinced to have communicated almost twice as long with patients and sevenfold with relatives than they did in reality. Further, they thought to need about three times longer for documentation than they really did. On the other hand, the physcians estimated their total working hours very realistically (Table 4).

**Table 4:** Comparison of measurement and self-assessment; rating of satisfaction in marks

	time/day (measurem.) [min]	time/day (self-assessm.) [min]	p (paired t-Test)	satisfaction (school marks)*	
				female	male
communication with patients	**79 min** [r: 32,23-147,47 min]	**133 min** [r**: 60-270 min]	0.00004	3.8	3.7
communication with relatives	**6 min** [r: 0-24,2 min]	**43 min** [r: 10-210 min]	0.00001	3.8	3.9
documentation	**148 min** [r: 22,18-157,91 min]	**226 min** [r: 60-360 min]	<0.00001		
working hours	**659 min** [r: 490-848 min]	**644 min** [r: 510-780 min]	>0.05		

*German school marks: 1 = very good, 2 = good, 3 = satisfactory, 4 = sufficient, 5 = poor, 6 = unsatisfactory

**r = range

### Satisfaction

Physicians' satisfaction with their communication was measured by using German school marks (1 = very good to 6 = unsatisfactory). The physicians graded their satisfaction with the time for communications with patients 3.7, satisfaction with communication with relatives was graded 3.8. We additionally calculated the Pearson correlation (including two-sided significance test) between the physicians' contentment and gender and also years of professional experience (Table 4). Over the course of their professional lives, the physicians' satisfaction with the time for communications with patients increased (p = 0.391), and their contentedness with the time for communications with relatives increased even significantly (p = 0.009). No gender differences could be found.

## Discussion

### Communication times with patients

Literature research revealed that studies on communication time between physicians and patients and between physicians and relatives, respectively, are rare. They are preferably done in the context of general practice consultations [7,8].

The present study is one of the first to look specifically into the physicians' time management on hospital wards and to research aspects of time in the communication with patients and relatives by systematic extrinsic measurements. Two studies which are comparable to our investigation researched communication times on ward rounds and were conducted in German hospitals, as well. In 1983, Fehrenberg et al. found an average time of 3.5 minutes per day for communication with patients on ward rounds. Fifteen years later Häuser and Schwebius calculated an average time of two minutes during which a physician communicates with each patient every day [9,10]. However, the times measured in both studies only count for conversations on ward rounds, and the data in the study of Häuser et al. are based on five extrinsic measurements only.

Available data show that physicians have only short time slots to communicate with patients in hospitals. Taking into account that during a clinical career spanning about 40 years, an oncologist, for example, is likely to conduct about 150.000 to 200.000 consultations with patients, communication should be viewed as a core clinical skill and an integral medical task [11]. From obtaining the patient's medical history to conveying a treatment plan, the physician's relationship with his patient is built upon communication. The actual discussion on the patient's autonomy and shared-decision-making is based on the possibility to build up a lasting and trusting relationship between patient and physician. With respect to this, the dialogue between the patient and physician is a prerequisite which must come into more focus again. To realise the patients' and physicians' demands on a medical system based on informed partnership rather than considerate paternalism, adequate time for patient-physician-dialogue will be a condition sine qua non.

Interestingly, although the communication times with patients were quite short, they have been overestimated by the doctors in our study. Multiple reports including our results reveal that physicians work load and multitasking demands are rather high [1,12-14]. Time pressure is a phenomenon commonly observed in clinical working fields which also influences communication times with patients. As Hemmer-Schanze et al. revealed three quarters of doctors have to shorten the dialogues with patients because of time pressure at least once a day [15]. Overestimation of communication time with patients by the doctors in our study may possibly be attributed to the feeling that communication with patients in the situation of time pressure is too time-consuming and that they might be swamped with their patients' complaints.

### Communication times with relatives

Up to our knowledge, there are hardly any examinations on communication times with relatives yet, except the study of Häuser et al. already mentioned above. Häuser et al. report a mean of 12.5 minutes total time for talks with relatives, which is about one minute per person a day. In the present study, time for communications with relatives has decreased to six minutes per day and physician, meaning a total time of 20 seconds for one patient's relatives a day [10]. How may this phenomenon be explained? An enormous workload and a growing number of administrative or documentation requests may contribute to the physicians' feeling that the demands of the patients' relatives are an additional burden to their work. The relatives and physicians' working times may further complicate frequent communication. Most people usually work until 5 or 6 o'clock in the afternoon and afterwards visit their relatives in hospital and want to talk to the doctor. At that time, physicians' regular work hours on ward are over as well, and communication with relatives, therefore, often must be done after end of work within their leisure time. Additionally, some relatives often ask to talk to the doctor without prior appointment. These inquiries interrupt the physicians' work and make them feel stressed and overwhelmed by multiple requests.

In an exploration on relatives' needs, Hartmann et al. found that 50% of relatives wish to accompany the physicians on their daily ward rounds; 21% would like to have family conferences together with physicians and the ill family member; 13% of relatives express a need of communication with the doctor on emotionally distressing issues [16]. Thus, communication with relatives has to be considered an integral part of clinical practice. Taking into consideration that relatives rate physicians' communication skills as important as their medical capabilities, the results of the present study should start further reflections on communication lengths and quality of dialogues with the relatives on hospital wards [17].

### The physicians' tasks on work

The time measurements demonstrated that the work load for physicians working on hospital wards is high. Breaks only accounted for 5.2% (35 minutes) of coded time in total which was much less than the official time allotted per shift for breaks. Time measurements also showed that the ward doctors were hard pressed for their time and tried to compensate for it by working overtime. An extra workload of about three hours a day was measured.

Looking at single items of time consumption, discussions with other specialists ranked first and accounted for 22.8% of coded time. Due to growing technical options medical examination methods are increasingly specialising and doctors have to discuss the results of examinations with specialists to be able to interpret and assess them.

Documentation including administrative requirements, DRGs (Diagnosis Related Groups) and others ranked second and took an average time of 148 minutes (2 hrs 28 min = 21.5% of work hours) per day. Administrative requirements take an increasing part of physicians' daily work and add to time pressure in clinical practice [3,10,13]. Doctors in the present study estimated that documentation would even take 35% of their working time. This overestimation may possibly be attributed to the feeling that administrative requirements are non-medical tasks and keep the doctors from doing their originally assigned work.

Our results regarding different work tasks are comparable to two recent studies focusing on the physicians' activities on hospital wards [18,19]. All studies showed that professional communication consumed the greatest proportion of working time whereas documentation tasks and patient care rank second. Further research is needed to qualitatively research the physicians' working tasks and identify the activities which could possibly be delegated to non-medical staff which would be less expansive and make physicians' work more effective [20].

### Physician satisfaction

The Physicians' level of satisfaction is linked to their perception of the amount of time that they have to perform their work [1]. Research revealed that the primary source of dissatisfaction of physicians is "time pressure" [13,21,22]. A sense of dissatisfaction and lack of time are known by many doctors in many countries [23]. Among other things, time pressure may be attributed to poor working conditions, tight appointment schedules, poor time management, legal conditions, Health Care System factors, lack of doctors working in hospitals or economic pressure [1,2,13]. The results reported in the present study show that the physicians' satisfaction either with their time for communicating with patients or with relatives is rather low. Physicians understand that the time spent with patients is a factor in patient satisfaction, too [24]. Consequently, in the long run neither the physicians nor the patients will be satisfied having to communicate on such a low scale.

According to the results of our study, the physicians seem to be more satisfied with their communication when they have already been working on their job for a longer period of time. The contentment with the time for communications with relatives was even significantly higher in our study after having worked professionally in this field for a considerable amount of time. These results are similar to the findings of other studies [25,26] and may be attributed to professional experience and greater range of communication skills. Possibly, physicians with longer experience either may have adjusted their expectations to the reality of the job or they may have improved their communication skills and need less time for valuable communications.

## Conclusions

More time for communication with patients and relatives on hospital wards is needed. Short doctor-patient communications make patient centred care more difficult which results in the physicians' dissatisfaction with their work (environment). As the present study was one of the first to research communication times and times for other medical tasks on hospital wards, further research is needed. Further research is also needed to assess the quality of communication with the patients and relatives on hospital wards. Communication time is only one aspect to look at of the important aspect of clinical communication. Another important issue will be to consider the physicians' communication skills. Physicians with successful communication skills will be able to handle communications more smoothly in situations of time pressure. Moreover, to provide patient centred, compassionate care, and to meet the needs of patients, relatives and physicians requires political and health care management solutions. Doctors should be allowed to focus on their core medical tasks and should have to spend less time on administrative requirements. Walkouts in German hospitals showed that unpaid overtime cannot be regarded as a solution for time pressure and excessive workload. The present study may be a wakeup call to initiate further steps to help foster physician-patient-communication by improving the working conditions on hospital wards. Our results in this respect may function as an impulse. But the results of this study should keep in mind the limitations of the methodology that was adopted here. The study used real time observation by a trained observer. As all measurements were done by one well trained medical student, a given standard in measurement procedures was guarantied. Nevertheless, there are likely to be some observer effects leading to possible differences to usual working patterns as doctors were followed and closely observed. Another distortion may be that the perspective of the measurement is the perspective of doctors' working time. Results of our study do not allow drawing any conclusions concerning the quality of communication with patients or relatives or quality of physician-patient relationships. Furthermore, no statements may be given on the patients' view on the physician-patient-communication.

## Competing interests

The authors declare that they have no competing interests.

## Authors' contributions

GB participated in study design and interpretation of data. She drafted the manuscript and contributed to all other aspects of the study.

DEK performed the external measurements and acquisition of data. CJX developed the idea and the design of the study. She also participated in the interpretation of the data.

FM made substantial contributions to the statistical analysis and helped to draft the manuscript. MO performed the statistical analysis. HEB made substantial contributions to interpretation of data and critically revised the manuscript.

## Pre-publication history

The pre-publication history for this paper can be accessed here:

http://www.biomedcentral.com/1472-6963/10/94/prepub
